# Humanitarian governance as an arena: (In)formal coordination and localised humanitarian action in Cúcuta, Colombia

**DOI:** 10.1186/s41018-026-00204-4

**Published:** 2026-09-15

**Authors:** Rodrigo Mena, Gabriela Villacis Izquierdo

**Affiliations:** International Institute of Social Studies, Erasmus University Rotterdam, The Hague, Netherlands

**Keywords:** Humanitarian governance, Humanitarian arena, Localisation, Everyday governance, Humanitarian-development-peace nexus, Mixed migration, Cúcuta, Colombia

## Abstract

This article examines how humanitarian governance in Colombia is shaped through the interaction of formal coordination mechanisms and informal, everyday practices. Using the humanitarian arena framework, alongside debates on localisation and the humanitarian–development–peace nexus, the article analyses humanitarian governance in Cúcuta, a border city affected by overlapping crises linked to armed conflict, migration, and structural inequality. Based on qualitative research including interviews, group discussions, and field observations, the study identifies a flexible and context-specific model of humanitarian governance that differs from conventional internationally led approaches. Findings show that humanitarian action in Cúcuta extends beyond emergency relief to include longer-term support for livelihoods, social protection, and community resilience. Humanitarian governance relies on both formal coordination structures and informal mechanisms, particularly *las mesas*, which facilitate negotiation, collaboration, and locally adapted responses. Nexus-oriented practices emerge organically from the realities of protracted crisis rather than through externally designed frameworks. The article contributes to debates on humanitarian governance by showing that negotiated humanitarian governance remains central even in contexts where state institutions retain significant regulatory authority. Rather than replacing formal governance arrangements, informal and everyday practices interact with them to shape humanitarian responses in complex crises.

## Introduction

In 2025, amid significant funding contractions and reductions in humanitarian programmes, the limitations of a highly internationalised humanitarian model have become increasingly evident. This model relies on external donors, United Nations (UN) agencies, and international NGOs (INGOs) operating through formal coordination mechanisms such as the Office for the Coordination of Humanitarian Affairs (OCHA) and the cluster system, designed to complement national and local authorities. Yet, as resources decline and the sector confronts structural constraints, less visible forms of humanitarian governance become increasingly important. These include the everyday practices, informal negotiations, and localised arrangements through which assistance is organised and delivered.

This article contributes to debates on humanitarian governance, localisation, and the humanitarian-development-peace nexus by examining how humanitarian governance operates in contexts where the affected state remains functional, centralised, and actively regulates humanitarian actors through national legal and institutional frameworks. Existing scholarship on the humanitarian arena has illuminated how aid is produced through negotiation among multiple actors with competing interests and logics (Fernando and Hilhorst 2006; Hilhorst and Jansen 2010; Knight, 2022; Witcher 2022). However, this body of work has paid comparatively limited attention to contexts where the state and local institutions retain strong regulatory authority, such as Colombia, where humanitarian actors operate within well-established legal and institutional frameworks.

In this article, we understand the humanitarian arena as the social and political space in which humanitarian action is negotiated among multiple actors -including state institutions, international organisations, local organisations, and crisis-affected populations- whose interests, priorities, and forms of authority often differ (Hilhorst and Jansen 2010; Hilhorst 2018a). We use the humanitarian arena as the analytical lens through which to examine how humanitarian governance in Cúcuta is configured through the interaction of formal coordination mechanisms and informal everyday practices, and how these negotiations shape humanitarian responses in practice.

Examining humanitarian governance through this lens allows us to address an important gap in the literature. While previous studies have highlighted the negotiated character of humanitarian action, less attention has been paid to how these negotiations unfold where state institutions retain significant regulatory authority and humanitarian actors operate within established legal and institutional frameworks. Rather, humanitarian action continues to be negotiated among state institutions, international organisations, local actors, and crisis-affected populations, even where legal frameworks and coordination mechanisms are well established. Drawing on the case of Cúcuta, this article demonstrates that negotiated humanitarian governance is not confined to contexts characterised by weak or absent state authority. Instead, humanitarian governance emerges through the interaction between comparatively strong state institutions and the informal, everyday practices through which actors adapt, negotiate, and implement humanitarian responses.

Using the case of Cúcuta, the capital of Norte de Santander department at the Colombian–Venezuelan border, we show that humanitarian governance in Cúcuta is shaped through the interaction of formal coordination systems and informal, localised practices. In this context of overlapping crises, humanitarian responses are continuously negotiated and implemented among diverse actors.

This analysis seeks to advance ongoing debates in three ways. First, while research on the humanitarian arena has convincingly demonstrated that aid is negotiated among diverse actors, it has devoted comparatively less attention to contexts where humanitarian governance unfolds alongside relatively strong state institutions and regulatory frameworks. This article addresses that gap by examining how legal authority and informal adaptive practices coexist and mutually shape humanitarian governance. Second, discussions on localisation have gained significant momentum since the 2016 World Humanitarian Summit^1^ and the Grand Bargain agenda, yet they remain centred on partnership models or funding transfers, with insufficient attention to the situated, relational practices through which localised governance is enacted. Third, although the humanitarian–development–peace nexus has become a central policy agenda, current understanding of how nexus approaches emerge organically, beyond donor-driven frameworks, remains limited. By tracing the ways in which actors in Cúcuta navigate and blend humanitarian, development and peacebuilding logics in practice, this article shows how informal mechanisms, coordination, and locally embedded governance practices configure humanitarian governance in ways that challenge and extend prevailing theoretical frameworks.

To explore these dynamics, the article draws on a qualitative case study in Cúcuta in 2023 and asks: How is humanitarian governance configured in Cúcuta, and how do formal and informal practices shape negotiation and coordination among state, international and local actors? To address this question, the article first outlines the context of Cúcuta and the overlapping crises shaping the humanitarian arena in the region. It then presents the theoretical framework guiding the study, followed by the methodology and findings. The final section discusses the broader implications of the analysis for debates on humanitarian governance under current global constraints.

## The case of Cúcuta, Colombia^2^  

Due to the convergence of multiple and simultaneous humanitarian crises, Colombia ranks first in the region for people in need of humanitarian assistance, ahead of Venezuela and Haiti (OCHA, 2025). In this article, we selected Cúcuta in Norte de Santander as a strategic case for examining humanitarian governance for several reasons, particularly its border location, the intensity of armed conflict dynamics, the presence of large migration flows, and recurring climate-related events (e.g., floods, landslides), which have created a diversity of overlapping crises (OCHA, 2026).

**Fig. 1:**
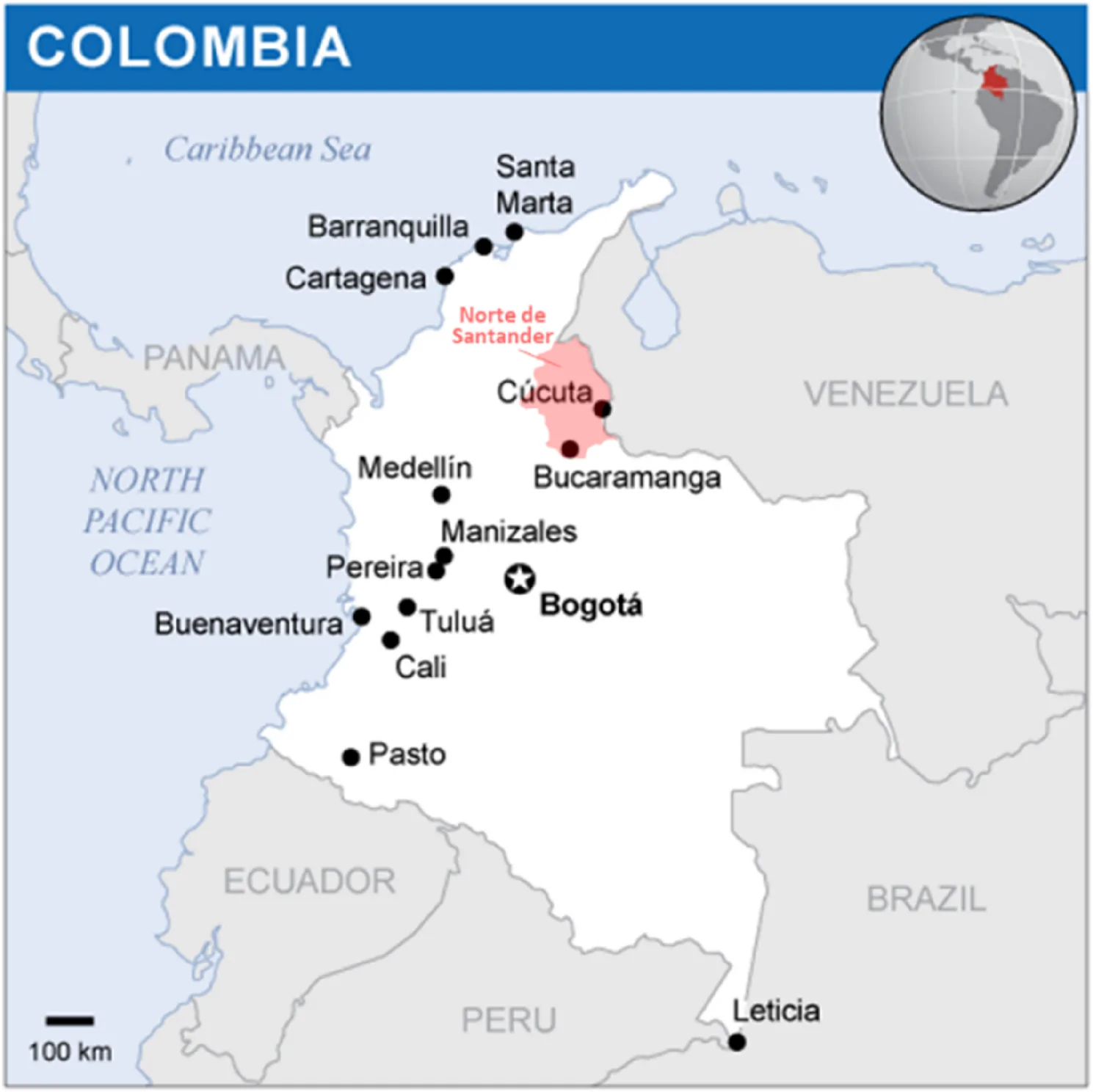
Map of Colombia. *Source*: OCHA/ReliefWeb, 2023 (https://reliefweb.int/location-maps)

Bordering Venezuela (see Fig. 1: Map of Colombia), Cúcuta has long been a hub of cross-border cultural and economic exchange. While trade spurred local industries, border fluidity has also facilitated illegal economies and the presence of irregular armed actors. The Colombia–Venezuela border is considered one of the most contested border regions in Latin America, marked by turf wars among multiple non-state armed groups (García Pinzón and Mantilla 2021). The resulting insecurity and the “governance” imposed by criminal organisations shape everyday life in the city (Mantilla 2023).

More broadly, the persistence of internal armed conflict in Norte de Santander, particularly in the Catatumbo region, continues to drive humanitarian crises and displacement across the department. Multiple armed actors –including state security forces, the National Liberation Army (ELN), dissident factions of the former Revolutionary Armed Forces of Colombia (FARC), mainly 33rd Front, paramilitary successor organisations such as the Ejército Gaitanista de Colombia (EGC, also known as Clan del Golfo), and criminal networks linked to transnational drug trafficking– contest territorial control and contribute to ongoing violence and insecurity on both sides of the Colombia–Venezuela border. According to the Local Coordination Team in Norte de Santander, these dynamics increasingly spill over into urban Cúcuta and surrounding rural areas (OCHA, 2026), where decades of conflict have displaced thousands of people, many of whom have settled in the city. The scale of this crisis is reflected in data from the Unit for the Attention and Integral Reparation to the Victims, which reports that 86% of registered victims of armed conflict in Norte de Santander have experienced forced displacement (UARIV 2025).

In addition, the deterioration of Venezuela’s sociopolitical and economic situation since 2015 has turned Cúcuta into a primary entry point for migrants and returnees (Ordóñez and Arcos 2019). Colombia hosts the largest number of Venezuelan migrants in the region—about 38% of all asylum seekers and migrants (Plataforma de Coordinación Interagencial para Refugiados y Migrantes (R4V) 2024). Humanitarian needs linked to Venezuela’s crisis have compounded existing inequalities. While Colombia has adopted relatively open migration policies (Beyers and Nicholls 2020), their implementation has been uneven, despite new measures such as the Comprehensive Protection Statute for Venezuelan Migrants (El Espectador, 2023). Current estimates suggest that Venezuelans comprise roughly 25% of Cúcuta’s population, putting additional pressure on urban services (Ordóñez and Arcos 2019). Therefore, the convergence of armed conflict, forced displacement, and mixed migration from Venezuela has positioned Cúcuta as one of the most complex humanitarian settings in the Americas.

### Main actors responding to humanitarian crises in Cúcuta

Humanitarian action in Cúcuta is shaped by a dense network of state institutions, international agencies, NGOs, faith-based organisations, and community actors responding to overlapping crises. Responses to armed conflict are coordinated primarily through local and national authorities, with support from OCHA and the Local Coordination Team (LCT), which brings together local and international humanitarian organisations. Since Venezuela’s crisis began, a large number of international agencies established operations in Cúcuta and are currently grouped under the Interagency Group for Mixed Migration Flows (GIFMM for its acronym in Spanish), which coordinate assistance for refugees, migrants, Colombian returnees, and the host population in coordination with the government since 2016. Policies such as CONPES 395 (2018) and CONPES 4100 (2022)^3^ have sought to strengthen institutional coordination and promote durable solutions for Venezuelans.

Cúcuta also hosts a large number of NGOs, faith-based groups, and community organisations that engage in humanitarian action, often without an explicit humanitarian mandate (Robillard et al. 2020). Local leadership structures, such as *Juntas de Acción Comunal (JACs)*^4^, have historically played an important role in community organisation, though rising urban violence and criminal threats have weakened these forms of participation (Sánchez and Gómez 2019). Despite these risks, local actors continue to organise, drawing on transnational solidarity and local forms of citizenship (Hochmüller 2022, p. 364).

Taken together, these overlapping dynamics –border fluidity, protracted armed conflict, mass displacement, and large-scale migration– make Cúcuta a uniquely revealing case for analysing humanitarian governance. The city embodies the convergence of national and transnational crises, where international and local actors operate side by side in conditions of insecurity, institutional fragmentation, and social inequality. It therefore offers a critical vantage point to understand how humanitarian responses are configured, negotiated, and adapted within a complex and contested environment. Studying Cúcuta allows us to examine humanitarian governance not as a fixed institutional design, but as a dynamic process shaped by everyday practices, informal networks, and the interactions between global frameworks and local realities.

## Humanitarian governance and cross-cutting topics

Complex humanitarian crises, such as those observed in Cúcuta, require a comprehensive approach that is informed by concepts that enable contextual research. The framework that informed our analysis began with ‘the humanitarian arena’ as a lens to examine humanitarian contexts through the complex and negotiated practices forming humanitarian action. Seeking to enrich our framework by including approaches that allow us to unveil the interplay of humanitarian action, development, and peacebuilding, the research also included perspectives on the triple nexus thinking and critical perspectives on localisation and localised humanitarian governance.

### Humanitarian governance as an arena

Building on the definition introduced above, the humanitarian arena serves as the analytical framework guiding this study. It enables us to examine how humanitarian action is shaped and negotiated in practice (Hilhorst 2018a), beyond its normative and formalised practices, and on how dynamic and complex interactions between diverse actors, including international organisations, NGOs, governments, and local communities, shape responses to the needs of populations affected by crises and disasters (Hilhorst and Jansen 2010). As Bakewell (2000, p. 104) poses, ‘[a]id programmes are not imposed on passive beneficiaries, but exist in an arena of social actors with competing interests and strategies.’ As such, the arena approach is an actor-oriented perspective, that recognises aid outcomes as negotiated through various social strategies, including coercion, formal interactions, and informal communication and mechanisms (Long 2001). These negotiations are based on the understanding that people can reflect on their experiences and interact strategically with others. The realities and impact of humanitarian actions are, therefore, shaped by how actors within and surrounding the humanitarian system interpret their context, needs, and roles (Melis and Apthorpe 2020; Mosse, 2013).

The arena perspective is part of a larger movement to recognise that humanitarian action has evolved from its original conception. Modern humanitarianism, often traced to Henry Dunant’s initiatives in the late 19th century, was originally described as the effort to ‘prevent and alleviate suffering, protect life, and ensure respect for the dignity of people in desperate situations as a result of conflict or disaster’ (Labbé and Daudin 2015, p. 186). Its primary focus was on responding to exceptional and localised crises (Barnett 2011; Davey et al. 2013). This model of ‘classic or traditional humanitarianism’ represents a paradigm in which aid is delivered based on principles and aimed at exceptional emergencies. However, over the course of its development, this has shifted. The internationalisation of humanitarianism, the persistence of protracted crises, and the growing recognition of diverse actors and interventions have led to the emergence of a new paradigm, often referred to as ‘resilience humanitarianism’ (Hilhorst 2018b). This approach shifts the focus from purely responding to immediate crises to building the long-term resilience of communities, emphasising sustainability and preparedness in the face of ongoing or future challenges.

Approaching humanitarian governance as an arena involves recognising its complex nature, its fluid connections, and its departure from classical humanitarianism towards resilience humanitarianism (Hilhorst 2018b). This perspective further acknowledges that humanitarian governance is not a fixed or static entity but rather a flexible process that involves the interplay of heterogeneous actors and practices, through which power relations and decision-making processes unfold (M. N. Barnett 2013; Gordon 2020; Hydén 2011). Local actors and community members play a crucial role in influencing these governance processes, highlighting the horizontal connections and multiple lines of influence within the arena, along which agency flows (Howes et al. 2015; Latour 2007; Pincock et al. 2021). In this article, therefore, humanitarian governance refers to the formal and informal processes through which humanitarian responses are coordinated, negotiated, and implemented among multiple actors.

An important feature of the humanitarian arena approach is its focus on everyday practices. Social interactions, contradictions, tensions, and negotiation are at the heart of the arena; and the context and the everyday realities of actors need to be acknowledged, as these translate into everyday politics (Artur 2011). With particular attention to the discourses and narratives of humanitarian actors and crisis-affected populations, a focus on everyday practices helps explain what is often unacknowledged in the formal structures of humanitarian ‘interventions’ and emergent forms of everyday governance (Cornea et al. 2017; de Herdt & Olivier de Sardan, 2015).

Drawing on the concept of everyday forms of real governance (Cornea et al. 2017; de Herdt & Olivier de Sardan, 2015; Titeca and de Herdt 2011), we understand governance as the practices and relationships through which decisions are negotiated and implemented in everyday life, rather than solely through formal institutions and official rules. From this perspective, humanitarian governance is not just about formal coordination, regulations, and structures; nor is it limited to moments of crisis but extends into the daily lives and less-formalised practices of affected people. In other words, humanitarian governance emerges through the everyday interactions and the (formal and informal) arrangements that emerge from those (Hilhorst and Jansen 2010; Long 2001).

### Localised humanitarian governance

An important outcome of the 2016 World Humanitarian Summit (WHS) was the promotion of strategies to transform humanitarian action into a more inclusive, non-hierarchical, and respectful arena towards local actors and local knowledges (World Humanitarian Summit Secretariat 2015). While calls for *localisation* predated the WHS, the summit was pivotal in advancing this agenda, particularly through the ‘Grand Bargain’, a commitment aimed at making humanitarian aid more efficient by channelling more power, funding, and decision-making to local and national actors (The Grand Bargain, 2016). By relying heavily on the commitments and perspective of international donors and agencies, the Grand Bargain claims to put affected populations and local organisations at the centre of humanitarian action, while at the same time, committing to allocate financial resources directly to them (Robillard et al. 2020).

These efforts are derived from the recognition that effective humanitarian responses require an active involvement and coordination with local actors, not only because they are usually the first respondents in a crisis (Rose and Jayawickrama 2016), but also because their knowledge, understanding of and proximity to the context are significant (Barakat and Milton 2020, p. 149). Consequently, with a broad and often contested definition, localisation refers to ‘the need to recognize, respect, strengthen, rebalance, recalibrate, reinforce or return some type of ownership or place to local and national actors’ (Barbelet, 2018, p. 5). Such an approach implies an intentional shift of humanitarian action centred around local actors.

However, the Grand Bargain process and ‘the turn toward localisation’ have been criticised, among other things, for a strong focus on funding matters, but mostly for failing to address the power dynamics embedded in the humanitarian arena (Peace Direct et al., 2021). Kuipers et al. (2020, p. 353) contend that policy and academic debates around localisation are still largely uninformed regarding three major issues: (i) Preference for local over nonlocal responses, as a dichotomy, without acknowledging the different local actors involved. (ii) The State is usually absent from the local debate; (iii) Different contexts are not acknowledged. Few of these analyses incorporate in practice studies of localisation or conflict-affected contexts. This is echoed by other scholars, who are concerned with aspects like the ‘practical implications and (im)possibilities of the localisation agenda, including shift of power relations and the conceptualisation of local itself’ (Melis and Apthorpe 2020, p. 367).

Considering these problematisations, the reduction of the localisation debate to a simple local vs. international binary is likely to contribute to the ‘exclusionary practices within the humanitarian sector’ (Pincock et al. 2021). Thus, the local is not static, homogeneous, or unified. In fact, the question of who and where the locals are is pertinent. Melis and Apthorpe (2020, p. 368) further problematise this by warning that such essentialisation could ignore the differences within the local and ‘neglect important questions regarding who is (and who is not) considered part of the local.’ By acknowledging the problematic assumptions and limitations of the term localisation, in this paper we instead use the term “localised humanitarian governance and action”. While localisation focuses on empowering local actors to take the lead in humanitarian responses, localised humanitarian governance and action emphasise the governance structures and processes that are specific to each context.

This approach can enable sensitive, participatory, and accountable humanitarian action at the level of (everyday) practices. This is an important caveat as a framework for this study, considering first the notion of the humanitarian arena, and secondly the perspective that in this article we examine the differences in humanitarian governance in Cúcuta as a distinct contextual approach, in contrast to international or more classic humanitarian approaches.

### (Triple) nexus thinking

From the perspective of the classical humanitarianism paradigm, there has been a traditional historical tendency to see development and humanitarian assistance as distinct processes, with humanitarian actions focusing on immediate aid during crises and development efforts seeking long-term systemic change to improve the well-being of communities (Brown et al. 2024; Frerks et al. 1999). Multiple initiatives and theories have emerged in recent years, all with the goal of better linking humanitarianism and development actions. Under the umbrella term of the ‘double nexus,’ these initiatives advocate for a more integrated and collaborative approach that bridges the gaps between different forms of assistance (Howe 2019; IASC, 2020). Examples of these developments include the ‘linking relief-rehabilitation-development’ (LRRD) approach, which aims to provide humanitarian assistance in a manner closely connected to medium and long-term development initiatives (VOICE NGO, 2006). Still, LRRD is not free from criticism for its linearity sequence and even deemed as a simplistic approach to complex situations (Christoplos and Hilhorst 2009).

More recently, this concept has evolved further, and since the WHS, there has been a growing recognition of the need to add peace to the double humanitarian-development nexus (OECD, 2021). First, there is a growing understanding that without peace, any form of aid may be undermined by conflict over time (Brown et al. 2024; Mena 2024). Second, the nexus perspective acknowledges that conflict and peace are not always separate realities, and many countries experience ongoing cycles of violent conflict and instability (Mena and Hilhorst 2022; Südhoff et al. 2020). This recognition highlights the importance of addressing not only immediate humanitarian needs but also the underlying causes of crises to achieve lasting peace and stability (CIC 2019; Steinke 2021).

In light of this, the WHS introduced the ‘new way of working’ agenda. This approach called for collaboration between humanitarian and development actors and emphasised integrating peace objectives to enhance aid effectiveness and align with the Sustainable Development Goals (OCHA, 2017, 2018). Known as the humanitarian-development-peace or triple nexus, this initiative spurred various publications, including the United Nations (UN) Secretary-General’s report ‘Sustaining Peace,’ 5 which underscores the need for collaborative efforts toward the 2030 Agenda for Sustainable Development, especially Sustainable Development Goal (SDG) 16 on peace, justice and strong institutions and SDG 4 on quality education (Schultze-Kraft 2022).

The triple nexus promotes a collaborative and holistic response, recognising connections of humanitarian, development, and peace challenges in complex crises (Brown et al. 2024). Its goal is to break down silos and strengthen coordination between these sectors for more sustainable and effective outcomes (Howe 2019). By addressing both immediate needs and the root causes of crises, the triple nexus aims to contribute to lasting peace and stability in conflict-affected regions. It emphasises coordination among humanitarian, development, and peacebuilding actors, recognising that progress in one area can positively impact the others (CIC 2019).

For this study, the triple nexus provides an analytical lens for understanding how humanitarian, development, and peacebuilding logics intersect in practice. Rather than assuming that nexus approaches are implemented through formal policy frameworks alone, we examine how actors in Cúcuta navigate and combine these different objectives in response to overlapping crises. This perspective complements the humanitarian arena framework by helping to explain how coordination and governance emerge across institutional boundaries and through everyday practice.

## Research methods

Based on the problem outlined in the introduction and the theoretical framework presented above, we used the case of Cúcuta to explore humanitarian governance dynamics in Colombia, guided by the following question: how is humanitarian governance configured in Cúcuta, and how do formal and informal practices shape negotiation and coordination among state, international, and local actors? In line with our conceptual framework, we focused on actors and their relationships through the following sub-questions: Who are the (local) humanitarian actors and subjects, and how are they defined as such? How are local and international frameworks intertwined, understood, and employed by humanitarian actors?

To answer these questions, we designed a qualitative case study combining literature review and fieldwork. The literature review included academic articles, book chapters, and institutional reports addressing humanitarian governance, coordination mechanisms, and localisation debates. We also examined grey literature related to Cúcuta and Colombia’s broader humanitarian architecture, including policy documents, evaluations, and situation reports produced by governmental bodies, international organisations, and NGOs. The review covered materials published from 2000 onwards, in both Spanish and English, to capture the evolution of humanitarian responses in the country and the specific dynamics of Cúcuta.

Following the literature review, we conducted fieldwork in Cúcuta in 2023. Participants were selected through a combination of purposive and snowball sampling. Purposive sampling guided the initial identification of key participants across four categories considered central to the research questions: representatives of Civil Society Organisations (CSOs) and NGOs (local, national, and international); UN agency managers and staff; local and municipal government representatives; and academic researchers with expertise in humanitarianism in Colombia. This strategy captured the diversity of actors involved in humanitarian action in Cúcuta.

Given the relational character of humanitarian coordination, snowball sampling was used to identify additional participants through referrals from initial interviews, which proved valuable for accessing actors operating in less formalised spaces. In total, we conducted eleven in-depth interviews and informal dialogues with fourteen participants, complemented by three group conversations. Table 1 provides an overview of the research participants.

**Table 1:** Research participants

Participant type	Number	Description
CSO & NGO representatives	4	Managers, country directors, and staff members of international, local, and national NGOs
UN managers and staff	3	Programme managers and directors
Government or municipality representatives	3	Government or municipality staff
Academics	4	Researchers working on humanitarianism in Colombia
Total	14	

Source: authors’ elaboration

These activities were complemented by in-situ observations across multiple sites and across Cúcuta, including border crossing points between Colombia and Venezuela, reception and transit areas where humanitarian services were being delivered, and neighbourhood-level spaces where NGOs operate. Observations focused on the spatial and material aspects of humanitarian organisations and service points. We also observed the interactions and relational dynamics among humanitarian actors and everyday practices in humanitarian spaces. After each observation episode, we systematically recorded our field notes and treated them as a distinct data source that we integrated into the thematic analysis and into our writing. The latter enriched our interview and group discussions material.

For data analysis, we applied thematic analysis structured around four predetermined themes, derived from the literature review and refined through the final theoretical framework. Codes were developed through an iterative process, combining deductive categories based on the conceptual framework with inductive insights emerging from the field data. The analysis was designed to address the research sub-questions: (1) perceptions of the drivers of humanitarian crises and the constitution of the humanitarian actors and subjects; (2) the relationship and complementarity between international and local frameworks for humanitarian response; (3) context-based understandings of the articulation between humanitarian action, development, and peacebuilding; and (4) formal and informal mechanisms of humanitarian governance. As the analysis progressed, additional themes that emerged from the data were incorporated to capture unanticipated but relevant dimensions of humanitarian governance in the case studied.

Regarding ethics and positionality, all authors are from Latin America, speak the local language, and have experience as researchers and practitioners in the humanitarian and development sectors. This positionality allowed for culturally and contextually grounded engagement, reflexivity, and trust-building during fieldwork. Consent was obtained from all participants, who were informed about the objectives of the research, their voluntary participation, and the confidentiality of their responses. Ethical considerations followed institutional research standards, including pseudonymization of data, secure storage of information, and avoidance of sensitive contexts, which were implemented to ensure the safety and integrity of all involved.

## Findings: understanding Cúcuta’s humanitarian governance arena

This section presents five key findings from our thematic analysis. The first subsection examines what humanitarian action entails in this context and how it is understood. The second subsection considers who the subjects of these actions are, while the third examines how such actions are governed and coordinated. Across these findings, nexus-oriented practices emerged as a cross-cutting dimension shaping both humanitarian action and governance.

The fourth key finding shows that humanitarian governance in Cúcuta operates between formal and informal practices, and between traditional humanitarian norms and locally negotiated arrangements that are adapted to context-specific realities and forms of coordination. The fifth subsection presents the case of Las Mesas as a clear example of this everyday, adaptive form of humanitarian governance.

### Broad Perceptions of Humanitarian Action

Our findings in Cúcuta unveil that humanitarian action is understood not as a narrow emergency response, but as a broad practice that addresses multiple crises. For most participants, humanitarianism encompassed both immediate relief from human suffering *and* longer-term efforts to support livelihoods, labour integration, and access to social services; areas not traditionally associated with humanitarian work. This expanded understanding reflects how local actors experience protracted and intersecting crises, where the boundaries between humanitarian, development, and social protection interventions are increasingly blurred.

Within this context, participants identified several sources of crisis beyond displacement and migration (often the main one depicted in the media), including the socioeconomic effects of the COVID-19 pandemic. Many described how the mixed migration flows from Venezuela added further vulnerabilities to the existing situation of people displaced by Colombia’s armed conflict, particularly those arriving from Arauca and Catatumbo, who had already established informal “rings of poverty” around the city. As multiple interviewees described, humanitarian responses in health, sanitation, and food security were already operating in these areas, but were further stretched by the compounded nature of the crises.

While this broader perception aligns with ideas of resilience humanitarianism, it also raises concerns about the capacity of actors to address such diverse and long-term needs. It underscores, as some aid actors interviewed indicated, the importance of ‘adaptive and flexible approaches’ that respond to shifting realities in the humanitarian arena, requiring organisations to reassess strategies, engage with local stakeholders, and integrate community perspectives. In this sense, humanitarian action in Cúcuta reflects resilience humanitarianism while also challenging the classical, short-term notion of emergency relief, raising the enduring question of *when humanitarianism becomes development* (Gabiam 2012).

As a result, humanitarian action can also be framed as solidarity, help, and even development, as framed during interviews, presented in one focus-group, and some texts (Aparicio 2015; Mena et al. 2022). Hence, participants in Cúcuta viewed humanitarian action beyond short-term relief as a means to strengthen community resilience and promote sustainable solutions, reflecting ideas advanced during the WHS and within the triple nexus approach. For most actors interviewed, the notion of “short-term humanitarianism” does not reflect their reality or way of working. As a representative of the Colombian Red Cross explained, the organisation adopts a comprehensive approach to crises before, during, and after crises, driven by the needs expressed by affected communities in Cúcuta and Norte de Santander. Their long-standing presence, knowledge of territorial dynamics, and close relationships with local populations make it impossible to confine their work to isolated projects or temporary interventions.

In this context, resilience humanitarianism is the *predominant* form of humanitarianism, in line with the arguments advanced by Hilhorst (2018). For instance, participants in one focus group with staff from an international NGO explained that organisational frameworks, donor expectations, and international priorities do not always correspond to local realities in Cúcuta.^6^ As a result, humanitarian actors often have to adapt interventions in practice, rely on local knowledge and experience, and go beyond formal guidelines in order to respond effectively. Participants also noted the need to strategically navigate how these adaptations are communicated back to donors and headquarters. These everyday adjustments produce forms of humanitarian governance that are negotiated and context dependent rather than fixed or standardised. Humanitarian governance in Cúcuta therefore unfolds through continuous mediation between institutional rules, local realities, moral considerations, and the agency of those implementing humanitarian action.

### *‘Do No Harm’ and the humanitarian subject in Cúcuta*

In defining who a humanitarian subject is and how humanitarianism unfolds, participants consistently emphasised a collective responsibility to *do no harm*, aligning with Anderson’s (1999) perspective that humanitarian assistance should avoid aggravating local conflicts and instead strengthen local capacities for peace. For actors embedded in formal coordination structures, this principle framed most of their actions and guided adherence to broader humanitarian norms. Civil society participants, meanwhile, invoked *do no harm* alongside ideas of solidarity and a moral duty to assist others. These locally grounded understandings resonate with the humanitarian principle of *humanity*, yet participants rarely articulated them in formal humanitarian terms. This suggests that their approach to humanitarianism is rooted in classical humanitarian values while also being shaped by everyday practice, moral worlds, and the contextual realities that legitimise certain ways of acting.

In line with the contextual understanding of humanitarianism discussed above, several participants highlighted that international cooperation, a term widely used in Colombia to refer to international development and humanitarian actors, was sometimes associated with actions that had caused harm to local communities. This perception was particularly linked to the initial humanitarian response to the “Venezuela situation,” where a lack of coordination and accountability undermined local trust. These early experiences, participants suggested, have since shaped how humanitarian actors define their roles, priorities, and target populations in Cúcuta. Furthermore, these actions can also be seen as discriminatory, as indicated by a UN humanitarian worker:

[…] a truck arrived loaded with aid, and people -Colombians- believed that it was for everyone, but they were told that it was only for migrants. In some cases, but not in all, it ended up causing harm […] people still say that certain humanitarian activities are more for migrants than for Colombians.’^7^

In addition, participants perceived harm as stemming from narrow understandings of aid provision. As discussed in one focus group, the provision of goods and cash transfers remains necessary, particularly in emergency contexts; however, over time the needs of affected populations evolve, and material assistance alone becomes insufficient if they are not accompanied by structural measures aimed at (labour market) integration and economic rights. A representative from one of the NGOs was critical of the way in which humanitarian organisations enhanced a form of ‘dependence on aid provision for some populations,’ in her words, limiting possibilities for sustainable solutions and integration.^8^

Such criticism also extends to other humanitarian responses and actors. Across observations, reviewed texts, and interviews, we were able to examine how the humanitarian arena in Cúcuta constantly adapts to context. Likewise, it was also possible to observe how the humanitarian subject can shift over time. In relation to humanitarian responses to armed conflict, one representative of a CSO was particularly critical of approaches promoted by international actors and the Colombian state, arguing that they often ‘cause harm and immobilise communities and social movements […] these actions lack a transformative perspective and are only aimed at survival.’^9^ In her view, tokenistic and welfare-oriented approaches reduce the agency of affected populations, a concern echoed by several other participants.

This understanding of the humanitarian subject and humanitarian action challenges traditional approaches to humanitarianism and its governance. In this case, accountability schemes also shift accordingly. *Downward accountability* (see Hilhorst et al. 2021) becomes more prominent, as responsibilities towards crisis-affected people and their ability to influence decisions gain central importance. This contrasts with *upward accountability*, which is typically directed towards donors and institutional authorities through reporting and compliance mechanisms. In addition, *sideways accountability*, meaning the obligations that humanitarian actors hold towards one another within a shared operational environment, plays a significant role in this context, as reflected in our findings and will be discussed in more detail below.

Organisations can hold one another accountable for maintaining limited views of the humanitarian subject and for how they decide what to act upon and how. This, in turn, also challenges coordination mechanisms. Moreover, this approach to the humanitarian subject and its role in a broader arena aligns more with a triple nexus approach, recognising that humanitarian actors can also be involved in development or other actions as needed, thus reducing donor influence in selecting target populations.

### Embracing the Nexus Approach

The broad scope of humanitarian subjects, needs and actions in Cúcuta required a more comprehensive form of humanitarian governance. In practice, actors in Cúcuta and Norte de Santander have developed an organic approach to governing the crisis that implicitly reflects a nexus approach, incorporating elements of development and peacebuilding into their responses. This approach has emerged naturally from the specific characteristics of the context and the need to fulfil multiple functions simultaneously. For example, the representative of the Colombian Red Cross in Norte de Santander indicated that their work is humanitarian when needed but goes beyond immediate responses when necessary. He pointed out that actions related to integral health and education are at the core of their mandate, together with peacebuilding efforts, mostly in terms of family reunification and search for missing persons in the context of armed conflict.^10^ As one UN staff shared,

In Norte de Santander, it should be clear that there are not only humanitarian actors, there are also the *cooperantes* [development cooperation actors] and some of them have mainly a humanitarian response, but they also have actions in peace and development, what is known as the famous triple nexus […] they started to realise that for a context like Norte de Santander […] they had to look for scenarios that favoured this transition […] once we overcome the humanitarian emergency, how do we give some tools to the affected people to have a dignified life?^11^

Despite this, our findings suggest that nexus-oriented approaches in Cúcuta were not primarily adopted through formal humanitarian frameworks or institutional directives. While participants occasionally used the term “nexus,” most were unfamiliar with the formal language and policy debates surrounding the “double” or “triple nexus.” Instead, these practices appeared to emerge pragmatically from the realities of the context itself. Faced with overlapping crises linked to displacement, migration, poverty, and violence, humanitarian actors described developing integrated responses because humanitarian needs could not be addressed in isolation from broader social, economic, and protection concerns. In this sense, nexus-oriented practices were less the result of top-down policy implementation than of everyday attempts to respond to interconnected vulnerabilities in practice.

While this nexus approach enables different actors to address root causes and promote sustainable solutions, it also introduces complexities in coordination and collaboration. As seen in the literature above, implementing nexus approaches requires continuous dialogue, adaptation, and coordination among diverse actors, which may lead to challenges in achieving effective coordination. The same challenges were observed and mentioned by the participants in Cúcuta. While these mechanisms and dialogues exist across formal and informal structures, they do not guarantee consensus or agreement. Moreover, the assumption that additional communication or discussion spaces (such as *‘las mesas*,’ to be explained later) can promote consensus and agreement should be approached cautiously, especially considering issues of diversity and power dynamics.

Despite these challenges, humanitarian actors in Cúcuta emphasise the necessity of the nexus approach to ensure complementarity and reduce duplication of efforts among various stakeholders. The integration of humanitarian, development, and peacebuilding efforts ‘allows actors to address emergency needs and take care of the poverty and other vulnerabilities, needed to have long term and real solutions,’ paraphrasing what two NGO interviewees indicated.^12^ However, the nexus approach also demands resource-sharing and collaboration, which may require navigating political and institutional challenges.

The possibilities for engaging beyond immediate crisis response was also pointed out by the representative of the local government, who explained that the administration is working towards adopting a more comprehensive framework to collaborate with UN agencies, INGOS, and NGOs. However, it was also shared about the political, legal, and financial constraints derived from the very nature of humanitarian action and the perception of the town hall as limited in its capacities: ‘For us, the humanitarian [work] is highly sensitive, the municipality has a very restricted humanitarian mission […] which is focused on disaster risks or victims according to the law.’^13^

Participants repeatedly highlighted the influential role of donors in shaping humanitarian action in Cúcuta. Although the context in Cúcuta requires more integrated and long-term approaches that connect development, peacebuilding and humanitarian action, participants argued that donor priorities often favour short-term projects with measurable and immediate results. Funding reductions and limited flexibility, partly linked to the prioritisation of other international crises, were widely perceived as major obstacles to developing more durable solutions. Participants noted that these constraints also shape how humanitarian governance is practised, limiting the ability of organisations to respond in more locally grounded and adaptive ways. At the same time, most interviewees emphasised the importance of more localised forms of humanitarian governance and action, which municipal authorities and United Nations actors alike argued must be rooted in and adapted to the specific realities of the context.

### Humanitarian governance between and within formal and informal coordination mechanisms

Theoretically, the humanitarian architecture in Cúcuta and Norte de Santander operates through structured and coordinated mechanisms, due to the existence of clusters and working groups on specific themes. In this sense, humanitarian action is formally organised through conventional governance and accountability structures. However, these structures were challenged once the so-called ‘Venezuela situation’ intensified in 2015–2016, as the existing responses and governance mechanisms were no longer considered effective.^14^ As a result, besides the LCT structure (in charge of humanitarian responses to the armed conflict and disasters in Norte de Santander), a new coordination group was created in 2016 to address the arrival of mixed migrants from Venezuela directly: the Interagency Group for Mixed Migration Flows (GIFMM for its acronym in Spanish). This coordination structure is unique to Colombia.

Both the LCT and the GIFMM in Cúcuta have clearly defined mandates. Still, they operate in parallel to provide joint responses in cases where situations of armed violence affect migrants or refugees from Venezuela, and they play a role in terms of sideways accountability. For the representative of the GIFMM in Norte de Santander the need for two separate mechanisms remains unresolved. However, it was indicated that this alternative form of localised humanitarian governance has proven effective in avoiding excessive operational and administrative burdens for a relatively small team, in this case, the local humanitarian coordinator and the staff.^15^

Humanitarian governance in Cúcuta extends beyond formal coordination structures and is also shaped through informal practices embedded in the everyday interactions of a diverse set of actors, including international NGOs, national NGOs, CSOs, state institutions, and migrant-led organisations. These actors do not participate on equal terms. Large international NGOs often occupy structurally dominant positions through their control over funding flows and programmatic priorities, while local NGOs and migrant-led organisations frequently have to negotiate space within these structures or operate at their margins (Armas and Freitez 2021). It is in the space between formal structures and everyday practice that power relations are most visibly negotiated.

Dialogue, negotiation and collaboration do not unfold on a level playing field. What occurs is that they reflect and reproduce hierarchies, even if sometimes they create space for local actors to contest, reframe, or redirect responses. Delivering humanitarian action through nexus-oriented or integrative approaches (bridging emergency relief, development programming and peacebuilding) intensifies these dynamics, requiring sustained communication and adaptive strategies across actors whose mandates, timelines and accountability structures frequently diverge. Formal mechanisms provide coordination spaces but rarely resolve underlying tensions. It is through informal negotiation that competing priorities are brokered, compromises reached and localised humanitarian governance is continuously reproduced.

Having such an informal and flexible approach is also something that international actors in other countries, who are attempting to implement a formal triple nexus framework, describe as challenging (Brown et al. 2024; Südhoff et al. 2020). Even so, people appear comfortable with this localised governance system, as they have long operated in this manner. Actors recognise the need for urgent responses while simultaneously engaging with medium- and long-term responses. In this context, governance mechanisms beyond formal coordination become essential. This flexibility enables actors to tailor their responses to local conditions and needs, fostering a sense of ownership and allowing for more rapid responses.

Interviews also suggest that this approach empowers local actors and communities to play an active role in shaping responses, strengthening their sense of agency and ownership over the humanitarian process. It also reinforces everyday accountability, as actors hold one another responsible, build trust and legitimacy, and collectively negotiate feasible ways to support people in need.

### Everyday humanitarian governance: The role of ‘las mesas’

 What shape does localised humanitarian governance take when humanitarian action is understood broadly, becomes nexus-oriented, and relies on less formal mechanisms? One way to answer this question is through the concept of *las mesas* (literally, “the tables”).

Las mesas, which can be loosely understood as working groups, play a central role in everyday humanitarian governance in Cúcuta. It is important to note that las mesas were not created by humanitarian actors nor specifically for humanitarian contexts. They have long been established in Colombia as key spaces for participation and coordination among communities, affected people, victims and government institutions. Las mesas are rooted in the National Constitution as an expression of participatory democracy. For example, national development plans are formulated through a participatory process in which different sectors of society convene in las mesas to present their demands and interests, which are then incorporated into the planning framework.

Because of this participatory function, las mesas have been adapted and replicated by a wide range of actors and sectors. Within humanitarian governance, one United Nations interviewee explained that

[In the GIFMM] below the plenary there are our ‘mesas,’ which are not clusters, they are technical ‘mesas’ that do not have a cluster structure, but they provide technical support to the decisions we make in the plenary. For example, if in the plenary it is said that we should build the school, the next thing to do is to go to the emergency education ‘mesa’ where the technicians meet and tell us how to build the school and so on.^16^

Navigating the numerous mesas within Cúcuta’s localised humanitarian governance presented notable challenges during the research, particularly identifying their existence, roles and interrelationships. The relatively limited formalisation of processes and documentation adds further complexity, making it difficult to understand their structures and to systematise their functioning. At the same time, this fluidity reveals much about how they operate beyond any structured or centralised system. This lack of clear road maps is not necessarily a weakness; as one participant suggested, it is also a strategy that enables adaptation and effective operation in a changing humanitarian context where flexibility and creativity are essential.^17^ A similar dynamic has been seen in Colombia regarding humanitarianism, particularly by civil society actors (see Aparicio 2015) indicating the flexibility of practices in the country when it comes to addressing crises.

More specifically, las mesas provide spaces for discussion, collaboration, and decision making among a wide range of actors, including affected communities and victims of armed conflict. In doing so, they strengthen accountability, although not without challenges, as we discuss later. These efforts to bring diverse voices into decision-making spaces have also strengthened coordination, particularly in comparison to the initial response to the Venezuelan crisis, which many participants described as fragmented and duplicative. For example, Decree 0376 of December 2022 created the *Mesa Migratoria*, which formalised discussions on the situation of mixed migrants, host communities and returnees within a single space. This coordination platform is led by the United Nations Refugee Agency (UNHCR) together with the Municipality of Cúcuta. The *mesa* convenes 18 local public institutions and regularly invites representatives from academia, migrant groups and other national institutions (Velásquez, n.d.). In practice, the aim of Mesa Migratoria is to contribute to the stabilisation and integration of mixed migrants through the collection of relevant data and needs assessment.

The less formal and dynamic character of these working groups fosters creativity and inclusivity, allowing diverse perspectives to address complex challenges. However, ensuring equitable participation, particularly for marginalised groups, remains difficult. Humanitarian actors in Cúcuta therefore work deliberately to strengthen inclusion and ensure that these voices inform decision-making.

Although mesas promote shared responsibility, their still limited formalisation can create gaps in accountability and coordination. Over time, however, many have developed stronger sideways and downward accountability practices, procedures and decision-making arrangements. The GIFMM, for instance, established its own accountability mesa. Participants consistently noted that these structures “developed over time”, incorporating elements of more formal humanitarian procedures while retaining the flexibility needed in the local context. As a result, mesas have become hybrid governance spaces that balance the expectations of international actors while remaining grounded in local needs and practices.

This integration of these accountability measures reflects attempts to strengthen transparency, coordination, and responsiveness within humanitarian governance practices. As one mesa coordinator noted, incorporating these working groups into a more coherent humanitarian governance framework is essential for strengthening coordination and ensuring their sustainability. In practice, accountability mechanisms within the LCT and the GIFMM operate largely through horizontal accountability with upward components, while INGO teams reported relying extensively on downward accountability strategies directed towards implementing partners and crisis-affected people.

Some interviewees expressed concern that, as certain mesas became more formalised and embedded in strategic governance structures, they also risked losing some of their initial flexibility and operational capacities. When this occurs, informal practices regain central importance: one-to-one conversations, shared coffee, lunches, or the creation of new mesas became regular practices for maintaining adaptive and responsive forms of coordination.

Such informal interactions are common across crisis settings worldwide, as noted in broader literature on informality in governance, including Mbaye and Dinardi’s (2019) work on cultural governance in Brazil and Senegal. What is distinctive in Cúcuta is that the mesas are not marginal to formal structures; from an analytical perspective they may appear as parallel governance arrangements, but for practitioners they are understood as integral to a localised humanitarian governance system that requires a high degree of flexibility. Such elements can be summarised in the testimony of one of the representatives of the local CSO:

once we identify the needs *with* the population, we establish the long-term plan to generate the transformation intervention, then we start the implementation and follow-up process and, at the end, the evaluation and validation […] by then we tell the community that we have finished the process […] then they are empowered in their processes and [the CSO] continues as a consultative body if they require additional support […] Who owns the process is the community.^18^

## Discussion and conclusion

The findings from Cúcuta’s humanitarian arena reveal a locally embedded model of humanitarian governance that operates across overlapping crises and institutional settings. The broad understanding of humanitarian action that characterises this arena enables actors to respond flexibly and operate across sectors (e.g., protection, livelihoods and integration as part of programmatic cycles). Yet this breadth creates its own tensions: organisations must simultaneously navigate protracted interconnected crises, competing institutional mandates, and shifting population needs, often under conditions of limited flexibility and constrained resources. In this context, adaptation relies on ongoing engagement with affected communities, allowing humanitarian actors to adjust responses as circumstances evolve.

Within this landscape, collaborative mechanisms such as *las mesas* emerge as important sites of humanitarian governance. Their semi-formal character creates spaces for participation and coordination, enabling actors to collectively navigate institutional constraints and context-specific challenges. At the same time, this fluidity requires deliberate effort to maintain transparency and accountability, particularly sideways accountability among peers. The Cúcuta experience also reveals persistent power asymmetries, where questions of inclusion and exclusion within decision-making spaces remain fundamental in a context marked by displacement, gendered inequalities, and uneven institutional authority.

The definition of the humanitarian subject is similarly shaped by context. While research participants identified armed conflict and migration as primary drivers of humanitarian need, they also emphasised structural conditions such as poverty, exclusion, and institutional neglect as equally important sources of vulnerability. In Cúcuta, this means that the boundaries of who counts as a humanitarian subject, and which crises warrant humanitarian response, are not fixed but continuously shaped by actors with different mandates, frameworks, and assessments of need. This speaks directly to Barnett’s (2013) observation that humanitarian governance is fundamentally a political process, one that involves the classification and management of populations in ways that carry important ethical implications. Given scarce resources, decisions about whom to assist are often driven as much by organisational and legal constraints and capacities as by need alone, producing forms of inclusion and exclusion with significant ethical and governance implications. This is precisely the terrain on which accountability becomes consequential: echoing Hilhorst et al. (2021, p. 365), responsibility involves not only holding others to account but also interrogating one’s own role in drawing (and redrawing) those boundaries.

Within this context and identified humanitarian subject, the triple nexus becomes particularly important. In Cúcuta, nexus framing is used by INGOs and state actors to justify integrated programming that bridges emergency relief, development and peacebuilding, reflecting the protracted nature of humanitarian crises. However, this does not occur without tensions, as Brown et al. (2024) caution. Nexus-oriented approaches can stretch organisational mandates and put pressure on core humanitarian principles such as independence and neutrality. For local NGOs and migrant-led organisations, a nexus logic is double-edged: it can open access to longer-term funding while imposing frameworks that may clash with community realities and organisational capacities.

Humanitarian actors in Cúcuta repeatedly highlighted the importance of reflexivity and an awareness that interventions can influence the lives and experiences of affected populations, emphasising the need for responses that are culturally appropriate, conflict-sensitive, and guided by the principle of doing no harm. Across the findings, this emphasis on reflexivity and contextual sensitivity is reflected in a humanitarian arena where legitimacy is not derived solely from formal mandates, but continuously constructed through dialogue and ongoing collaboration. It is within this arena that decisions are made, and localised humanitarian governance takes shape. Formal mechanisms are not discarded; rather, they are continuously reinterpreted and intertwined with the informal practices through which humanitarian action is sustained.

This model offers both opportunities and challenges. While integrating humanitarian action with local governance systems can enable more holistic and sustainable responses, it also brings into focus persistent constraints related to unstable funding, uneven policy coherence, and unequal forms of representation. More broadly, the Cúcuta case extends humanitarian arena scholarship by showing that negotiated humanitarian governance is not limited to settings characterised by weak or fragmented state authority. Instead, our findings demonstrate that negotiation remains central even where state institutions retain considerable regulatory capacity and humanitarian action operates within established legal and institutional frameworks. In such contexts, informal governance does not replace formal institutions; rather, it complements, reshapes, and at times challenges them through the everyday interactions of humanitarian actors.

In this way, the Cúcuta case contributes empirically to broader scholarly debates that conceptualise humanitarian governance as a relational arena shaped by power relations, informality, and multiple, sometimes competing, forms of accountability (Gordon 2020; Hilhorst et al. 2021). It also speaks to the literature on everyday governance (Cornea et al. 2017; Titeca and de Herdt 2011), demonstrating that the micro-level practices through which actors build trust, manage competing priorities, and navigate institutional constraints constitute governance. While grounded in a specific borderland setting, the dynamics identified here are likely to resonate with other contexts characterised by protracted displacement, layered governance arrangements, and diverse humanitarian practices.

This analysis has limitations that point toward future research directions. By adopting an organisational and institutional perspective, it does not fully examine how affected populations themselves understand and navigate the humanitarian systems and practices, or how they experience formal and informal governance structures. Further research is required to explore whether the dynamics identified here align with, or diverge from, the lived experiences and perspectives of affected populations in Cúcuta.

In sum, Cúcuta illustrates how humanitarian governance emerges through the interaction of formal and informal practices, nexus-oriented approaches, and diverse accountability relations among actors occupying different positions, mandates, and interests. Rather than confirming humanitarian governance as either institutionally driven or primarily informal, the findings demonstrate that governance is continuously produced through the interaction of formal institutions and everyday negotiated practices. The Cúcuta case therefore suggests that the humanitarian arena should be understood not only as a feature of fragile or weak governance environments, but also as an analytical lens for understanding humanitarian action in contexts where relatively strong state institutions coexist with informal and adaptive forms of coordination. Ultimately, the Cúcuta case demonstrates that humanitarian governance is sustained not only through formal institutions, but through the everyday practices and relationships that make coordination possible.

## Data Availability

No datasets were generated or analysed during the current study.

## Abbreviations

CSO: Civil Society Organisation
GIFMM: Interagency Group for Mixed Migratory Flows
INGO: International Non-Governmental Organisation
JAC: Junta de Acción Comunal
LCT: Local Coordination Team
LRRD: Linking Relief–Rehabilitation–Development
NGO: Non-Governmental Organisation
OCHA: United Nations Office for the Coordination of Humanitarian Affairs
UNHCR: United Nations Refugee Agency
UARIV: Unidad para la Atención y Reparación Integral a las Víctimas
UN: United Nations
WHS: World Humanitarian Summit

## Footnotes

1 The WHS was global event held in Istanbul in 2016. It was organised by OCHA after the initiative of the UN Secretary General, Antonio Guterres, and focused on reforming humanitarian aid to enhance efficiency and collaboration. A total of 9,000 participants from 180 member states attended the summit.

2 Our research is contextual to the time when we conducted fieldwork and analysis (April 2023- June 2024). Therefore, we acknowledge the latest events of massive forced displacement and armed violence in Catatumbo intensifying in late 2024 and 2025

3 CONPES (Consejo Nacional de Política Económica y Social) is Colombia’s highest national planning authority, responsible for issuing policy guidelines and directing public investment across sectors.

4 A Junta de Acción Comunal (JAC) is a grassroots community organisation, legally recognised in Colombia, through which residents of a neighbourhood or rural area collectively address local needs, liaise with authorities, and manage community development initiatives.

5 The Sustaining Peace agenda became a central priority at the beginning of UN Secretary-General António Guterres’ tenure, building on the 2016 UN General Assembly and Security Council resolutions on sustaining peace and reinforcing integrated approaches to humanitarian action, development, and peacebuilding.

6 Group discussion with humanitarian workers in Cúcuta, April 12, 2023.

7 Interview 1 with UN staff member in Cúcuta, April 5, 2023.

8 Interview 9 with local CSO representative in Cúcuta, April 11, 2023.

9 Interview 7 with local CSO representative in Cúcuta, April 11, 2023.

10 Interview 5 with Colombian Red Cross representative in Cúcuta, April 10, 2023.

11 Interview 4 with UN staff member in Cúcuta, April 10, 2023.

12 Group discussion with humanitarian workers in Cúcuta, April 12, 2023.

13 Interview 2 with municipality representative in Cúcuta, April 5, 2023.

14 Interview 3 with UN staff member in Cúcuta, April 5, 2023 and Group discussion with humanitarian workers in Cúcuta, April 12, 2023.

15 Interview 3 with UN staff member in Cúcuta, April 5, 2023.

16 Interview 3 with UN staff member in Cúcuta, April 5, 2023.

17 Group discussion with humanitarian workers in Cúcuta, April 12, 2023.

18 Interview 9 with CSO representative in Cúcuta, April 11, 2023.
